# Oral Microbiome Signatures in Periodontitis and Edentulism—A Population‐Based Study

**DOI:** 10.1111/jre.70046

**Published:** 2025-11-01

**Authors:** Preethi Balan, Fabio R. M. Leite, John Rong Hao Tay, Jeffry Hartanto, Gustavo G. Nascimento, Mario Romandini

**Affiliations:** ^1^ National Dental Research Institute Singapore National Dental Centre Singapore Singapore City Singapore; ^2^ Oral Health Academic Clinical Program Duke‐NUS Medical School Singapore Singapore City Singapore; ^3^ School of Dentistry University of Utah Salt Lake City Utah USA; ^4^ Department of Restorative Dentistry National Dental Centre Singapore Singapore City Singapore; ^5^ Health Services and Systems Research Programme Duke‐NUS Medical School Singapore City Singapore; ^6^ Ninth People's Hospital, Shanghai Jiao Tong University School of Medicine Shanghai China

**Keywords:** Defluviitaleaceae_UCG‐011, bacteria, biofilm, dysbiosis, epidemiologic factors, etiology, inflammation, microbiology, pathogenesis, periodontal diseases, saliva

## Abstract

**Aim:**

To examine the association between the oral microbiome, periodontitis, and edentulism in a nationally representative sample of the U.S. population.

**Methods:**

A total of 5299 adults aged 30–69 years were examined (NHANES 2009–2012). Oral rinse samples were collected and analyzed through 16S rRNA gene sequencing. Periodontitis presence, stage, extent, and grade were assessed according to the 2017 AAP/EFP classification using the ACES framework, with edentulism considered as a distinct category. Bacterial diversity and taxonomic composition were evaluated using alpha and beta diversity metrics and multivariable linear models (MaAsLin2), adjusted for relevant confounders.

**Results:**

Alpha diversity increased with periodontitis severity, extent, and grade, peaking in Stage III generalized periodontitis. In Stage IV, extensive tooth loss was associated with a decrease in alpha diversity. Edentulous individuals exhibited the lowest alpha diversity, falling below levels observed in those without periodontitis. Beta diversity differences across periodontitis severity, extent, and grade were subtle (< 0.2%). Taxonomically, increasing severity, extent, and grade of periodontitis were associated with enrichment of established periodontitis‐related genera (e.g., *Dialister*, *Filifactor*, *Fusobacterium*, *Porphyromonas*, *Prevotella*, *Tannerella*) and *Jonquetella*, alongside depletion of health‐related genera (e.g., *Rothia*, *Veillonella*). A total of 13 genera were commonly altered in both edentulous individuals and those with Stage III–IV periodontitis, relative to participants with no or localized Stage I–II disease.

**Conclusion:**

Periodontitis is characterized by an increase in alpha diversity with advancing severity, extent, and grade, followed by a decline with extensive tooth loss and edentulism. However, it accounted for only a small fraction of the overall variation in oral microbiome composition. Taxonomic shifts included enrichment of established periodontitis‐related genera and *Jonquetella*, alongside depletion of health‐related genera. The persistence of periodontitis‐associated bacteria in edentulous individuals may have important implications for implant dentistry.

## Graphical abstract

This study represents the first population‐based investigation of the oral microbiome in relation to periodontitis and edentulism. Periodontitis is characterized by a paradoxical increase in alpha diversity with advancing severity, extent, and grade, followed by a decline with extensive tooth loss and edentulism. Taxonomic shifts included enrichment of established periodontitis‐related genera (e.g., *Dialister*, *Filifactor*, *Fusobacterium*, *Porphyromonas*, *Prevotella*, *Tannerella*) and *Jonquetella*, alongside depletion of health‐related genera (e.g., *Rothia*, *Veillonella*).

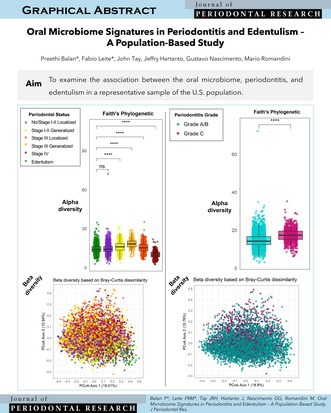

**Summary**

- Background
  ○ Despite its central role in the pathogenesis of periodontitis, existing studies on the oral microbiome largely rely on convenience samples. Moreover, most investigations have focused narrowly on the subgingival biofilm, rather than assessing the oral microbiome as a whole. An additional unresolved question is how the oral microbiome of edentulous individuals compares to that of individuals with periodontitis.
- Added value of this study
  ○ This study represents the first population‐based investigation of the oral microbiome in relation to periodontitis and edentulism. Periodontitis was characterized by a progressive increase in alpha diversity as disease stage, extent, and grade advanced, with only subtle shifts in beta diversity. Taxonomically, increasing severity, extent, and grade of periodontitis were associated with enrichment of established periodontitis‐related genera and *Jonquetella*, alongside depletion of health‐related genera. Notably, 13 genera were commonly altered in both edentulism and Stage III–IV periodontitis.
- Clinical implications
  ○ The paradoxical increase in within‐sample microbial diversity with advancing periodontitis stage, extent, and grade—despite only subtle shifts in overall microbial composition—raises important questions about the disease's etiopathogenesis. Furthermore, the persistence of periodontitis‐associated taxa even in the absence of teeth may have significant implications for implant‐based rehabilitations and the prevention of peri‐implant diseases.

## 1 Introduction

Periodontitis is a highly prevalent disease resulting from complex and incompletely deciphered imbalances between the oral microbiome and the host immune response [1, 2, 3, 4], facilitated by various systemic and environmental factors [5, 6, 7, 8, 9, 10, 11, 12, 13]. Despite the microbiome's central role in both the pathogenesis of periodontitis and its association with systemic diseases [14, 15, 16, 17, 18, 19, 20], available studies rely on convenience samples [21, 22, 23, 24, 25, 26, 27], introducing a potential for selection bias. Furthermore, the majority of investigations have narrowly focused on the subgingival biofilm [21, 22, 23, 24, 25, 28, 29], rather than analyzing the oral microbiome as a whole [26, 27]. This site‐specific approach contrasts with the current conceptualization of periodontitis as a systemic disease [30]. Another unresolved question concerns how the oral microbiome of edentulous individuals compares to that of individuals with periodontitis. This has important implications not only for understanding the disease etiopathogenesis but also for guiding clinical decision‐making, particularly in the context of dental implant rehabilitations and the prevention of peri‐implant diseases [31, 32]. To address these knowledge gaps, the present study aimed to examine the association between the oral microbiome, periodontitis, and edentulism in a nationally representative sample of the U.S. population.

## 2 Methods

This manuscript adheres to the Strengthening the Reporting of Observational Studies in Epidemiology (STROBE) [33]. The study followed the PECOS framework:

(P) *Participants*: Representative sample of the civilian, noninstitutionalized U.S. population (NHANES 2009–2012).

(E) *Exposure*/(C) *Comparison*: Oral microbiome profiles, assessed via 16S rRNA gene sequencing.

(O) *Outcomes*: Periodontal status (defined by the intersection of periodontitis presence, stage, and extent, with edentulism considered as a distinct condition), periodontitis grade, and number of sites with probing pocket depth (PPD) ≥ 6 mm.

(S) *Study Design*: Cross‐sectional.

Further methodological details are provided below.

### 2.1 Population

The population for this cross‐sectional study was sourced from the 2009–2012 cycles of the National Health and Nutrition Examination Survey (NHANES 2009–2012). Conducted by the U.S. Centers for Disease Control and Prevention (CDC), NHANES employs a stratified, multistage, clustered probability sampling design to produce a nationally representative sample of the U.S. civilian, noninstitutionalized population [34]. Detailed descriptions of the survey content and sampling methodology are available elsewhere [35]. The NHANES protocols were approved by the CDC's National Center for Health Statistics Research Ethics Review Board, and all participants provided written consent form.

Individuals were included in the present analysis if they were aged 30–69 years, had undergone oral microbiome sampling, and received both a dental and periodontal (if not edentulous) examination.

### 2.2 Exposure and Comparison: Oral Microbiome

Oral rinse samples were collected from study participants using a standardized protocol [36]. Specifically, participants were instructed to rinse with 10 mL of Scope mouthwash (Procter & Gamble, Cincinnati, Ohio, USA) or saline for 30 s, alternating between swishing and gargling every 5 s. The rinse was then expectorated into a sterile collection cup prior to oral and periodontal examinations.

Genomic DNA was extracted from the oral rinse samples following a prespecified protocol [37]. After centrifugation, samples underwent sequential digestion with DNase‐free RNase A and proteinase K. DNA was purified using the Qiagen Virus/Bacteria Midi Kit with the Pathogen Complex 800 program on the QIAsymphony SP instrument (Qiagen) and eluted in 60 μL of AVE buffer. Each sequencing batch included blank samples and two artificial community samples (oral and gut) for quality control. Extracted DNA was shipped on dry ice to the Knight Laboratory at the University of California, San Diego, and stored at −20°C until sequencing.

Detailed protocols for DNA sequencing and bioinformatic processing are available elsewhere [38]. In brief, the V4 region of the 16S rRNA gene (2 × 125 bp) was amplified via polymerase chain reaction (PCR) and sequenced on the Illumina HiSeq 2500 platform, following manufacturer guidelines. Demultiplexing was performed using QIIME v1.9. Due to the lack of overlap between paired‐end reads, only forward reads were used for analysis. Sequence processing was conducted using the DADA2 pipeline (version 1.2.1), and taxonomic assignment was performed using the SILVA v123 database. As this was a 16S rRNA gene‐based analysis, only bacterial taxa were assessed.

Microbiome metrics included:

- *Alpha diversity*: Observed amplicon sequence variants (ASVs), Faith's phylogenetic diversity, Shannon–Wiener index, and inverse Simpson index, extracted at a rarefaction depth of 10 000 reads with 9 iterations.
- *Beta diversity*: Bray–Curtis dissimilarity, unweighted UniFrac, and weighted UniFrac distances. ASV feature tables were rarefied to 10 000 reads per sample. Phylogenetic trees were used to compute UniFrac distances. Bray–Curtis dissimilarity was calculated independently of phylogeny.
- *Taxonomic composition*: Relative abundance and read counts at the genus level. In the present study, significant genera identified from the SILVA v123–based analysis were subsetted and mapped using labels from the Human Oral Microbiome Database (HOMD) [39].

### 2.3 Outcomes: Periodontal Status, Periodontitis Grade and Number of PPD ≥ 6 mm

The oral health examination was performed by trained dental hygienists (NHANES 2009–2010) and licensed general dentists (NHANES 2011–2012). Tooth count was assessed first. In participants with at least one natural tooth, a full‐mouth periodontal examination was performed using a color‐coded periodontal probe (PCP2, HuFriedy). Measurements of gingival margin position and PPD were recorded at six sites per tooth (excluding third molars, partially erupted teeth, and retained roots where the CEJ and part of the clinical crown were not present). Clinical attachment level (CAL) was subsequently calculated from these measurements. Examiners were trained and calibrated prior to the survey and re‐calibrated 2–3 times annually. Additional details on the periodontal examination procedures are available elsewhere [35].

Three outcomes were considered for this analysis: (i) periodontal status, (ii) periodontitis grade, and (iii) number of sites with PPD ≥ 6 mm. Primary teeth were not considered. Participants were excluded if they had PPD or CAL data missing in one or more quadrants despite having permanent teeth, or if they had fewer than two nonadjacent teeth (rendering periodontal status classification unfeasible).

Due to the limited number of “no periodontitis” cases (*N* = 1), periodontal status was categorized into the following groups, based on the intersection of periodontitis presence, stage, and extent, with edentulism considered as a distinct category:

1. No periodontitis or Stage I‐II periodontitis, localized (reference group)
2. Stage I‐II periodontitis, generalized
3. Stage III periodontitis, localized
4. Stage III periodontitis, generalized
5. Stage IV periodontitis
6. Edentulism

Edentulism was defined as the complete absence of natural teeth. Periodontitis was defined as the presence of either [40]:

- Interdental CAL ≥ 1 mm at ≥ 2 nonadjacent teeth, or
- Buccal/lingual CAL ≥ 3 mm with PPD > 3 mm at ≥ 2 nonadjacent teeth.

Periodontitis stage and extent were determined using the ACES framework [41, 42]. Due to data limitations, furcation involvement was not included in the staging process.

Periodontitis grade was also assessed using ACES criteria, based on the tooth showing the highest percentage ratio between the worst interproximal CAL and the corresponding root length, using established reference root lengths [43]. Grade modifiers (i.e., diabetes and smoking) were not applied, as these variables were considered confounders in the present analysis. Given the low number of grade A cases (*n* = 57), periodontitis grade was dichotomized as A/B (reference group) versus C.

PPD was categorized into three groups based on the total number of sites with PPD ≥ 6 mm in the whole dentition: 0 sites, 1–4 sites, and ≥ 5 sites [44].

### 2.4 Confounders

The following key confounders were considered: age, gender, ethnicity, education, smoking status, diabetes status, body mass index (BMI), number of teeth, antibiotic use, and presence of untreated caries (Directed Acyclic Graph shown in Figure S1). Ethnicity was categorized as Non‐Hispanic White, Non‐Hispanic Black, Mexican American, other Hispanic, and other or Multi‐Ethnicity. Education was classified into three levels: high school diploma or less, some college or Associate of Arts degree, and college graduate or above. Smoking status was categorized as never (fewer than 100 cigarettes in a lifetime), former, or current smoker, based on self‐reported data [45]. Diabetes status was categorized by HbA1c levels into: no diabetes (< 5.7%), prediabetes (5.7%–6.4%), optimally controlled diabetes (6.5%–6.9%), fairly controlled diabetes (7.0%–8.9%), and poorly controlled diabetes (≥ 9.0%) [46, 47]. Participants were classified by BMI as underweight (< 18.5 kg/m^2^), normal weight (18.5–24.9 kg/m^2^), overweight (25.0–29.9 kg/m^2^), or obese (≥ 30.0 kg/m^2^) [48]. Antibiotic use was coded as a binary variable (yes/no) based on self‐reported prescription medication use in the past 30 days. The presence of untreated caries was classified as a binary variable (yes/no), based on the presence of at least one tooth with an untreated carious lesion [49].

### 2.5 Data Analysis

All statistical analyses were conducted using R (version 4.4.3), with statistical significance set at *p* < 0.05. Three analytic frameworks were employed, considering either “periodontal status”, “periodontitis grade”, or “number of sites with PPD ≥ 6 mm” as the outcome.

Alpha diversity indices were first compared across groups using the Wilcoxon rank‐sum test. Subsequently, linear regression models were fitted to adjust for potential confounders.

Beta diversity was evaluated using Permutational Multivariate Analysis of Variance (PERMANOVA) with 999 permutations. To assess differences in dispersion, Permutational Analysis of Multivariate Dispersions (PERMDISP) was conducted, followed by pairwise comparisons using Tukey's Honest Significant Difference (HSD) test.

Associations between microbial genera and periodontal outcomes were analyzed using Multivariate Association with Linear Models (MaAsLin2), applying default settings. Genus‐level relative abundance analyses were restricted to genera with a prevalence of at least 1% (*n* = 170). An UpSet plot was generated to visualize intersections of differentially abundant genera across the three outcomes, and a heatmap (produced via the *pheatmap* package) illustrated the relative abundance patterns of overlapping taxa.

Sensitivity analyses were performed. Survey‐weighted generalized linear models (*svyglm*) were used to examine associations between alpha diversity metrics and periodontal outcomes accounting for the complex sample design, with predictive margins estimating adjusted means. Similarly, for beta diversity, survey‐adjusted PERMANOVA was conducted using the *fast.adonis* function with 999 permutations and 100 bootstrap resamples to support distance‐based multivariate testing. Survey‐adjusted analyses were not performed for taxonomic abundance due to current methodological limitations; most available tools are indeed not compatible with complex survey designs and face convergence issues when modeling sparse, zero‐inflated microbiome data. Instead, a sensitivity analysis of taxonomic composition was performed using the original SILVA v123 database assignment.

All analyses were adjusted for the previously described confounders, with the exception of the Wilcoxon rank‐sum tests and PERMDISP analyses, which were unadjusted. Multiple testing correction was applied using the Benjamini‐Hochberg procedure to control the false discovery rate.

## 3 Results

Of the 20 293 individuals enrolled in NHANES 2009–2012, 14 456 were excluded due to age restriction (*n* = 12 545), missing dental and/or periodontal examinations (*n* = 490), or lack of oral microbiome sampling (*n* = 1421). An additional 538 participants were excluded for either lacking PPD/CAL data in one or more quadrants despite having permanent teeth (*n* = 531) or having fewer than two nonadjacent permanent teeth (*n* = 7). As a result, the final analytical sample included 5299 participants.

Table 1 summarizes the characteristics of the study population. Overall, 4.4% of participants were edentulous, while 95.5% had periodontitis (corresponding to > 99.9% of the dentate population), including a 16.0% prevalence of Stage IV disease and 16.4% with Grade C periodontitis.

**TABLE 1:** Characteristics of the study population.

Characteristic		Overall (*N* = 5299)
Age, mean (SD)		48.53 (11.2)
Gender, *N* (%)	Female	2642 (49.9)
	Male	2657 (50.1)
Ethnicity, *N* (%)	Non‐Hispanic White	2061 (38.9)
	Non‐Hispanic Black	1185 (22.4)
	Mexican American	887 (16.7)
	Other Hispanic	565 (10.7)
	Other or Multi‐Ethnicity	601 (11.3)
Education, *N* (%)	≤ High school diploma	2487 (46.9)
	Some college or AA degree	1455 (27.5)
	≥ College graduate	1357 (25.6)
Smoking status, *N* (%)	Never smoker	2907 (54.9)
	Former smoker	1214 (22.9)
	Current smoker	1178 (22.2)
Diabetes status, *N* (%)	No diabetes	3046 (57.5)
	Prediabetes	1628 (30.7)
	Optimally controlled diabetes	200 (3.8)
	Fairly controlled diabetes	254 (4.8)
	Poorly controlled diabetes	171 (3.2)
Body mass index, *N* (%)	Underweight	53 (1.0)
	Normal weight	1291 (24.4)
	Overweight	1850 (34.9)
	Obese	2105 (39.7)
Antibiotic use in the previous 30 days, *N* (%)	No	5135 (96.9)
	Yes	164 (3.1)
Number of teeth, mean (SD)		23.81 (7.8)
Presence of untreated caries, *N* (%)	No	4844 (91.4)
	Yes	455 (8.6)
Periodontal status, *N* (%)	No/Stage I–II Localized	1419 (26.8)
	Stage I–II Generalized	1696 (32.0)
	Stage III Localized	894 (16.9)
	Stage III Generalized	209 (3.9)
	Stage IV	846 (16.0)
	Edentulous	235 (4.4)
Periodontitis grade,^a^ *N* (%)	Grade A/B	4229 (83.5)
	Grade C	834 (16.5)
PPD ≥ 6 mm,^a^ *N* (%)	0 sites	4307 (85.1)
	1–4 sites	502 (9.9)
	≥ 5 sites	254 (5.0)

Abbreviations: AA, Associate of Arts; *N*, number; PPD, probing pocket depth; SD, standard deviation.

^a^ The total *N* is reduced (*N* = 5063) due to the exclusion of edentulous individuals (*n* = 235) and participants without periodontitis (*n* = 1).

### 3.1 Oral Microbiome Shifts Across Periodontal Status, Including Edentulism

**FIGURE 1:**
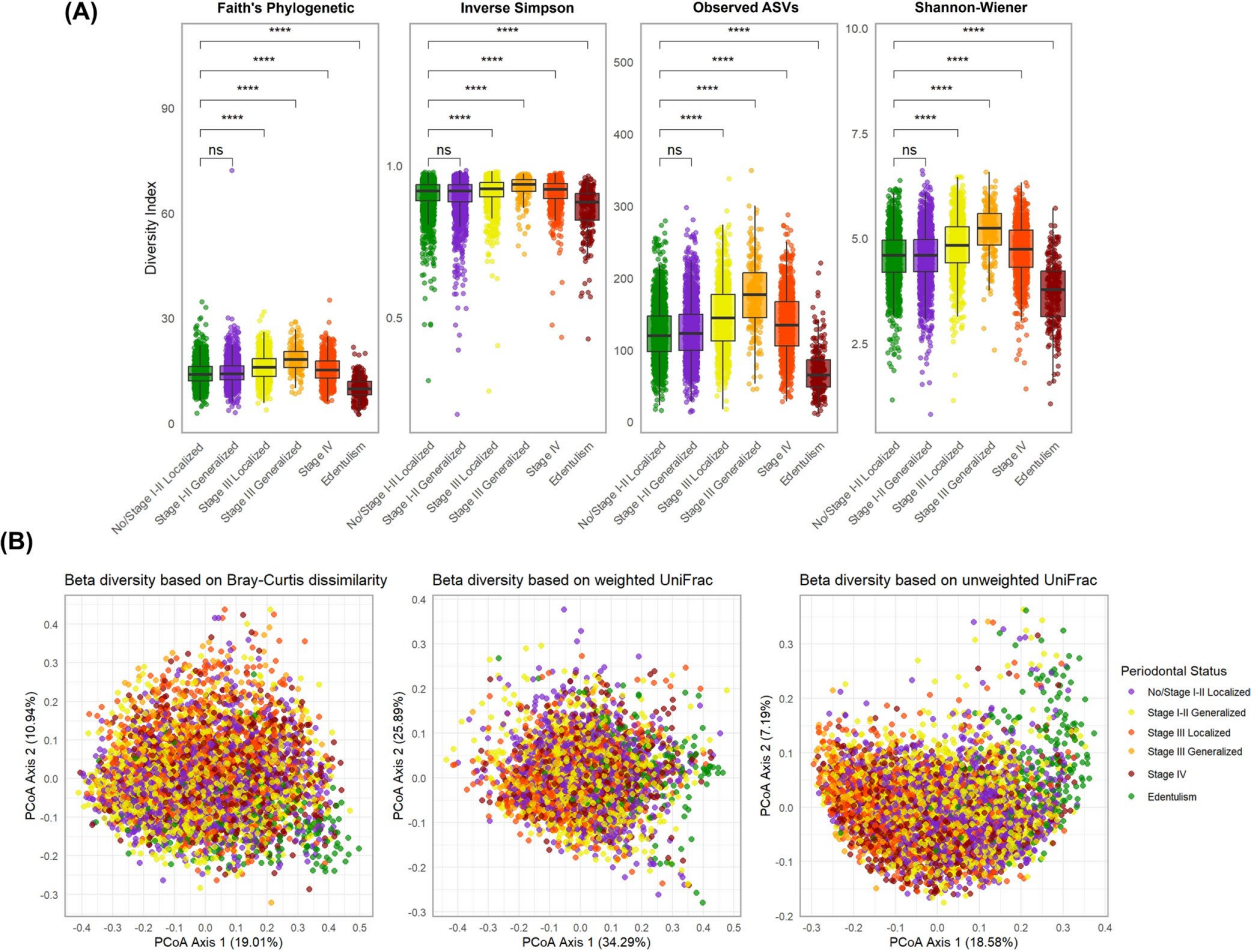
Alpha and beta diversity across different “periodontal status” categories. (A) Alpha diversity indices. Box plots illustrate the interquartile range (IQR) from the 25th (Q1) to the 75th percentile (Q3), with the centerline indicating the median (Q2). Jittered points represent individual data points to visualize distribution density. Whiskers extend to 1.5× the IQR, and points beyond these are plotted as outliers. (****, *p* < 0.0001 after FDR correction; ns, nonsignificant). (B) Beta diversity indices. Principal Coordinates Analysis (PCoA) plots display the proportion of variance explained by the first two principal coordinates (PCo1 and PCo2), as indicated on the axes. Each point represents an individual sample, and spatial proximity indicates higher similarity in microbial community composition.

Alpha diversity across the different periodontal status categories is reported in Figure 1A and Table 2. In unadjusted analyses (Figure 1A), compared to the reference group—individuals with no periodontitis or Stage I–II localized periodontitis—median alpha diversity increased progressively with advancing disease stage and extent, peaking in Stage III generalized periodontitis. However, individuals with Stage I–II generalized periodontitis did not show a statistically significant difference in alpha diversity relative to the reference group. Although Stage IV periodontitis was associated with higher alpha diversity than the reference, values were lower than those observed in Stage III generalized cases. Edentulous individuals showed the lowest alpha diversity overall, even below that of the reference group. Results remained consistent after adjustment for confounders (Table 2) and in sensitivity analyses using survey weights (Table S1).

**TABLE 2:** Alpha diversity indices across different “periodontal status”, “periodontitis grades”, and “number of sites with PPD ≥ 6 mm”.

Alpha Diversity Index	Periodontal outcome	Estimate	Standard error	*p*
Faith's Phylogenetic Diversity	No/Stage I–II Localized	*Reference*		
	Stage I–II Generalized	0.218	0.122	0.121
	Stage III Localized	1.449	0.151	**< 0.001**
	Stage III Generalized	3.467	0.259	**< 0.001**
	Stage IV	1.973	0.188	**< 0.001**
	Edentulism	−1.334	0.371	**0.001**
	Grade A/B	*Reference*		
	Grade C	2.289	0.137	**< 0.001**
	0 sites with PPD ≥ 6 mm	*Reference*		
	1–4 sites with PPD ≥ 6 mm	2.007	0.164	**< 0.001**
	≥ 5 sites with PPD ≥ 6 mm	2.892	0.226	**< 0.001**
Inverse Simpson	No/Stage I–II Localized	*Reference*		
	Stage I–II Generalized	0.000	0.002	0.886
	Stage III Localized	0.007	0.003	**0.024**
	Stage III Generalized	0.020	0.005	**< 0.001**
	Stage IV	0.014	0.003	**< 0.001**
	Edentulism	−0.025	0.007	**0.001**
	Grade A/B	*Reference*		
	Grade C	0.013	0.002	**< 0.001**
	0 sites with PPD ≥ 6 mm	*Reference*		
	1–4 sites with PPD ≥ 6 mm	0.014	0.003	**< 0.001**
	≥ 5 sites with PPD ≥ 6 mm	0.016	0.004	**< 0.001**
Observed ASVs	No/Stage I–II Localized	*Reference*		
	Stage I–II Generalized	2.403	1.397	0.124
	Stage III Localized	17.536	1.730	**< 0.001**
	Stage III Generalized	44.441	2.965	**< 0.001**
	Stage IV	24.849	2.158	**< 0.001**
	Edentulism	−15.353	4.246	**0.001**
	Grade A/B	*Reference*		
	Grade C	27.986	1.584	**< 0.001**
	0 sites with PPD ≥ 6 mm	*Reference*		
	1–4 sites with PPD ≥ 6 mm	22.966	1.898	**< 0.001**
	≥ 5 sites with PPD ≥ 6 mm	36.050	2.607	**< 0.001**
Shannon–Wiener	No/Stage I–II Localized	*Reference*		
	Stage I–II Generalized	0.022	0.023	0.411
	Stage III Localized	0.191	0.029	**< 0.001**
	Stage III Generalized	0.511	0.049	**< 0.001**
	Stage IV	0.309	0.036	**< 0.001**
	Edentulism	−0.317	0.070	**< 0.001**
	Grade A/B	*Reference*		
	Grade C	0.329	0.026	**< 0.001**
	0 sites with PPD ≥ 6 mm	*Reference*		
	1–4 sites with PPD ≥ 6 mm	0.286	0.031	**< 0.001**
	≥ 5 sites with PPD ≥ 6 mm	0.418	0.042	**< 0.001**

*Note:* Bold indicates statistical significance (*p* < 0.05).

Beta diversity findings across periodontal status categories are reported in Figure 1B and Table 3. PERMANOVA analyses indicated subtle (< 0.2%), mostly nonsignificant (with the exception of unweighted UniFrac), compositional differences across all periodontal status groups compared to the reference. PERMDISP analyses showed that within‐group dispersion tended to slightly increase in Stage IV periodontitis (weighted UniFrac only) and in edentulism (except for unweighted UniFrac). Sensitivity survey‐weighted PERMANOVA analyses revealed greater magnitudes of compositional differences across periodontal status categories, with variance explained reaching up to 1.6% (Table S2).

**TABLE 3:** Beta diversity across different “periodontal status”, “periodontitis grades”, and “number of sites with PPD ≥ 6 mm”.

Beta diversity metric	Periodontal outcome	PERMANOVA		PERMDISP			
		*R* ^2^ (%)	*p*	Dispersion difference	Lower CI	Upper CI	*p*
Bray Curtis Dissimilarity	No/Stage I–II Localized	*Reference*		*Reference*			
	Stage I–II Generalized	0.026	0.666	0.004	−0.006	0.014	0.807
	Stage III Localized	0.068	0.129	0.007	−0.005	0.018	0.604
	Stage III Generalized	0.124	0.099	0.007	−0.013	0.028	0.906
	Stage IV	0.084	0.099	0.011	−0.001	0.023	0.104
	Edentulism	0.054	0.575	0.023	0.003	0.042	**0.011**
	Grade A/B	*Reference*		*Reference*			
	Grade C	0.130	**0.011**	0.006	−0.001	0.013	0.071
	0 sites with PPD ≥ 6 mm	*Reference*		*Reference*			
	1–4 sites with PPD ≥ 6 mm	0.045	**0.036**	0.005	−0.006	0.015	0.558
	≥ 5 sites with PPD ≥ 6 mm	0.038	0.055	0.000	−0.014	0.015	0.998
Weighted Unifrac	No/Stage I–II Localized	*Reference*		*Reference*			
	Stage I–II Generalized	0.046	0.278	0.002	−0.006	0.010	0.975
	Stage III Localized	0.093	0.148	0.007	−0.002	0.017	0.254
	Stage III Generalized	0.151	0.147	0.015	−0.002	0.032	0.117
	Stage IV	0.151	0.065	0.015	0.005	0.025	**< 0.001**
	Edentulism	0.012	0.973	0.018	0.002	0.032	**0.016**
	Grade A/B	*Reference*		*Reference*			
	Grade C	0.133	**0.011**	0.005	−0.001	0.011	0.097
	0 sites with PPD ≥ 6 mm	*Reference*		*Reference*			
	1–4 sites with PPD ≥ 6 mm	0.062	0.066	0.006	−0.003	0.015	0.227
	≥ 5 sites with PPD ≥ 6 mm	0.037	0.184	0.003	−0.009	0.015	0.837
Unweighted Unifrac	No/Stage I–II Localized	*Reference*		*Reference*			
	Stage I–II Generalized	0.045	0.140	−0.001	−0.010	0.008	0.999
	Stage III Localized	0.139	**0.010**	−0.004	−0.014	0.007	0.906
	Stage III Generalized	0.185	**0.010**	−0.003	−0.021	0.015	0.999
	Stage IV	0.099	**0.020**	0.005	−0.006	0.015	0.798
	Edentulism	0.069	0.297	0.008	−0.009	0.025	0.797
	Grade A/B	*Reference*		*Reference*			
	Grade C	0.101	**0.011**	0.002	−0.004	0.007	0.592
	0 sites with PPD ≥ 6 mm	*Reference*		*Reference*			
	1–4 sites with PPD ≥ 6 mm	0.043	**0.015**	−0.001	−0.010	0.007	0.944
	≥ 5 sites with PPD ≥ 6 mm	0.050	**0.015**	0.009	−0.003	0.020	0.208

*Note:* Bold indicates statistical significance (*p* < 0.05).

Abbreviation: CI, 95% confidence intervals.

**FIGURE 2:**
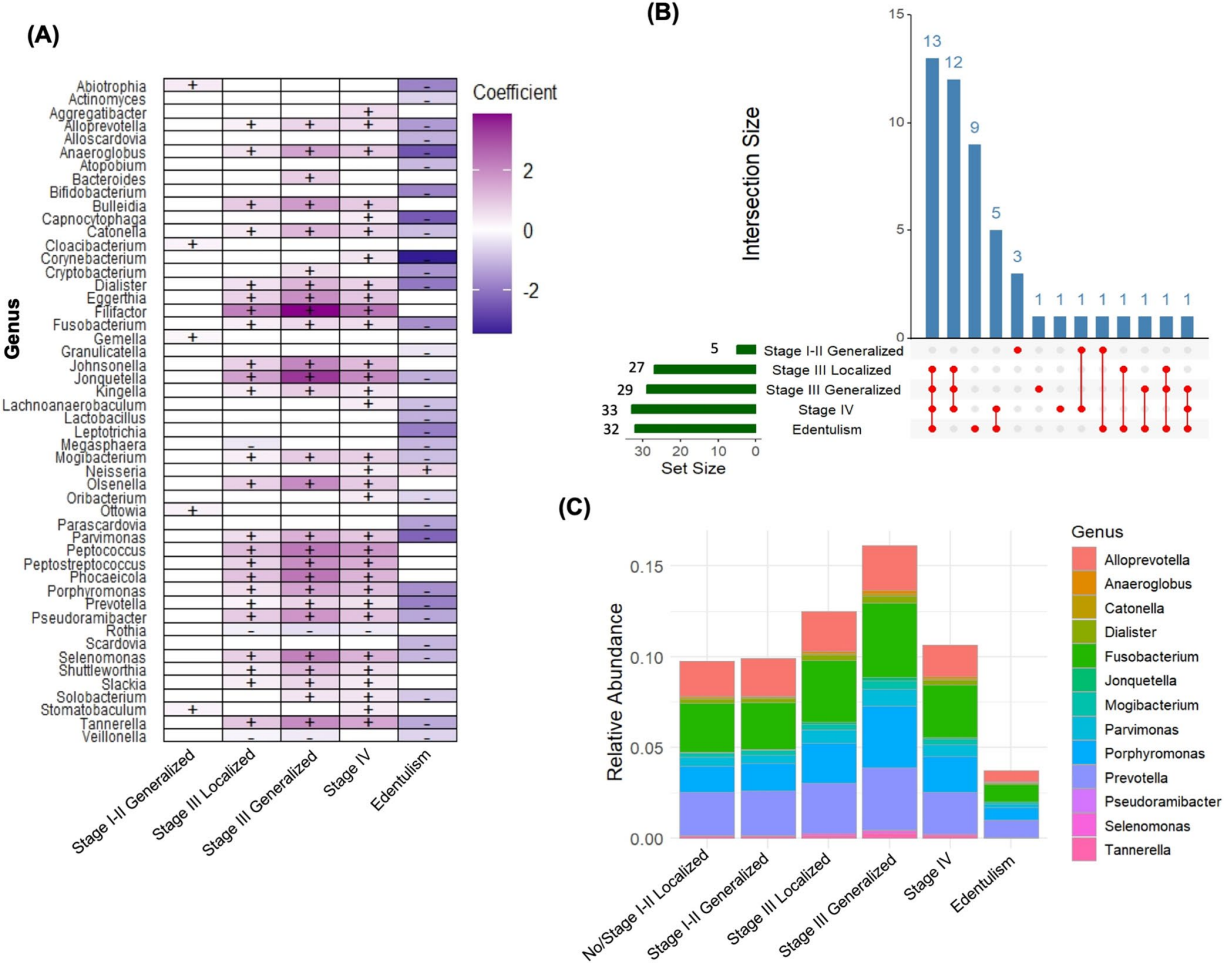
Differentially abundant taxa across different “periodontal status” categories (based on HOMD‐matching). (A) Differential abundance analysis using MaAsLin2. The heatmap presents genera significantly associated with each periodontal status compared to the reference group (no periodontitis/Stage I–II localized). Effect sizes indicate the strength and direction of associations; “+” denotes positive and “–” negative associations (*p* < 0.05 after FDR correction). (B) UpSet plot of differentially abundant genera. The horizontal bars indicate the total number of significantly altered genera in each periodontal status group. The vertical bars display the number of genera shared across combinations of groups. (C) Heatmap of overlapping genera. This heatmap illustrates the relative abundance of genera that were commonly identified as differentially abundant across multiple periodontal status categories.

MaAsLin2 analysis results based on HOMD conversion are shown in Figure 2. Advanced stages of periodontitis were positively associated with classical periodontitis‐related genera, such as *Dialister*, *Filifactor*, *Fusobacterium*, *Porphyromonas*, *Prevotella*, and *Tannerella*, but also with *Jonquetella*. Conversely, health‐related early colonizers such as *Rothia* and *Veillonella* showed negative associations (Figure 2A). Only five differentially abundant genera were detected in Stage I–II generalized periodontitis compared to no/Stage I–II localized periodontitis (Figure 2B). In contrast, more substantial shifts emerged from Stage III onward: 27 differentially abundant genera in Stage III localized periodontitis, 29 in Stage III generalized, and 33 in Stage IV. Among edentulous individuals, 32 genera were differentially abundant compared to the reference group—31 of which showed decreased abundance. Notably, 13 genera were commonly altered in the edentulous group and individuals with Stage III–IV periodontitis. Figure 2C illustrates the relative abundance of genera across periodontal status categories. Total HOMD‐based microbial load steadily increased from the reference group to Stage III generalized periodontitis, then declined in Stage IV to levels between Stages II and III, and dropped sharply in the edentulous group. Total HOMD‐based microbial abundance in edentulous individuals was approximately one fourth of that observed in Stage III generalized periodontitis. In the broader SILVA‐based sensitivity analysis, the results were largely consistent; however, additional genera, such as *Defluviitaleaceae_UCG‐011*, were also associated with advanced stages of periodontitis (Figure S2).

### 3.2 Oral Microbiome Shifts Across Periodontitis Grades

**FIGURE 3:**
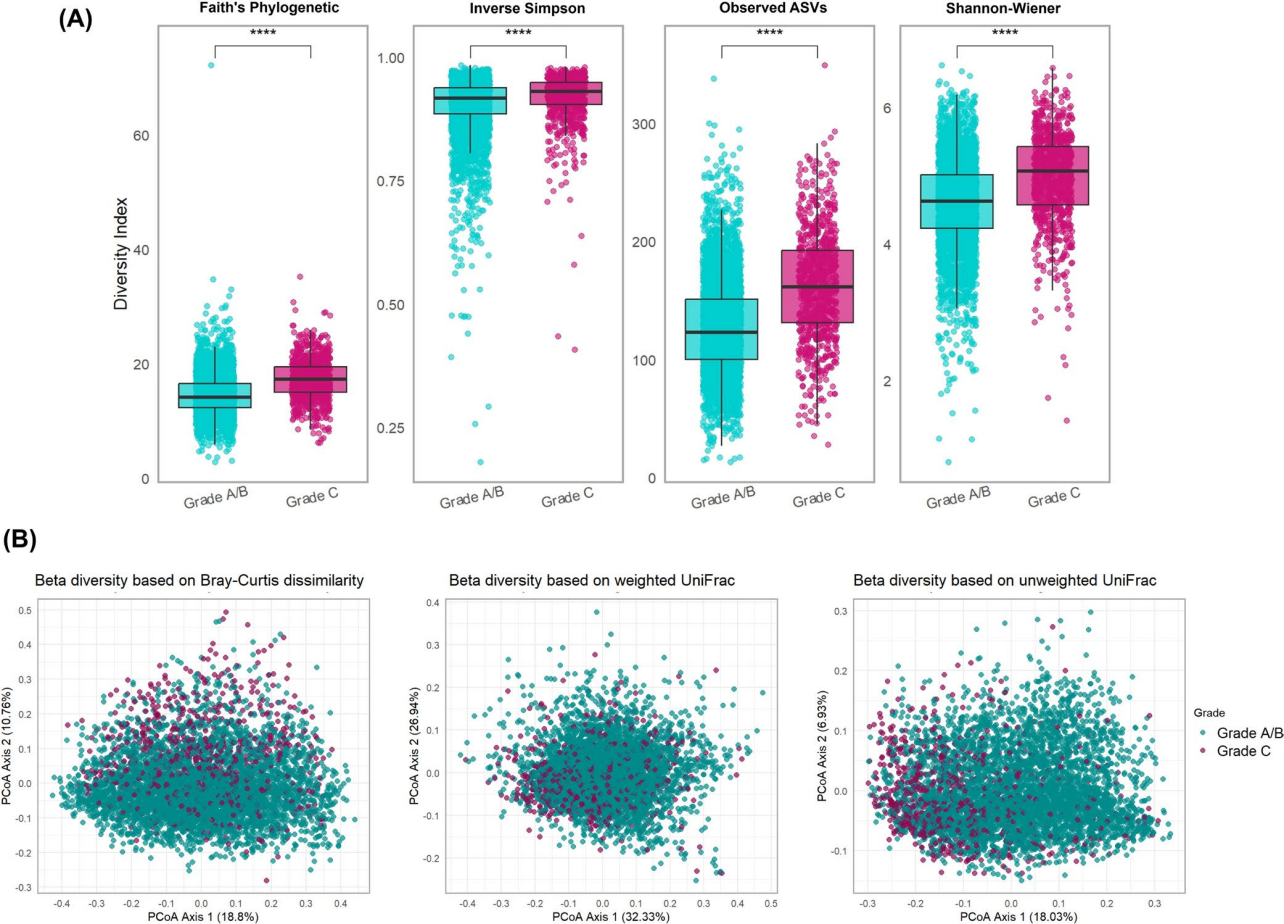
Alpha and beta diversity across different “periodontitis grades”. (A) Alpha diversity indices. Box plots illustrate the interquartile range (IQR) from the 25th (Q1) to the 75th percentile (Q3), with the centerline indicating the median (Q2). Jittered points represent individual data points to visualize distribution density. Whiskers extend to 1.5× the IQR, and points beyond these are plotted as outliers. (****, *p* < 0.0001 after FDR correction). (B) Beta diversity indices. Principal Coordinates Analysis (PCoA) plots display the proportion of variance explained by the first two principal coordinates (PCo1 and PCo2), as indicated on the axes. Each point represents an individual sample, and spatial proximity indicates higher similarity in microbial community composition.

A significant increase in alpha diversity was observed in Grade C compared to Grades A/B, in both unadjusted and adjusted analyses (Figure 3A and Table 2). PERMANOVA revealed statistically significant, though modest (0.1%), differences in beta diversity between the grades, while PERMDISP indicated no significant differences in dispersion across any metric (Figure 3B and Table 3). Sensitivity survey‐weighted analyses yielded consistent results for alpha diversity (Table S1), while beta diversity analyses revealed stronger magnitudes: considering weighted UniFrac, Grade C explained 2.29% of the variance compared to Grades A/B (Table S2).

**FIGURE 4:**
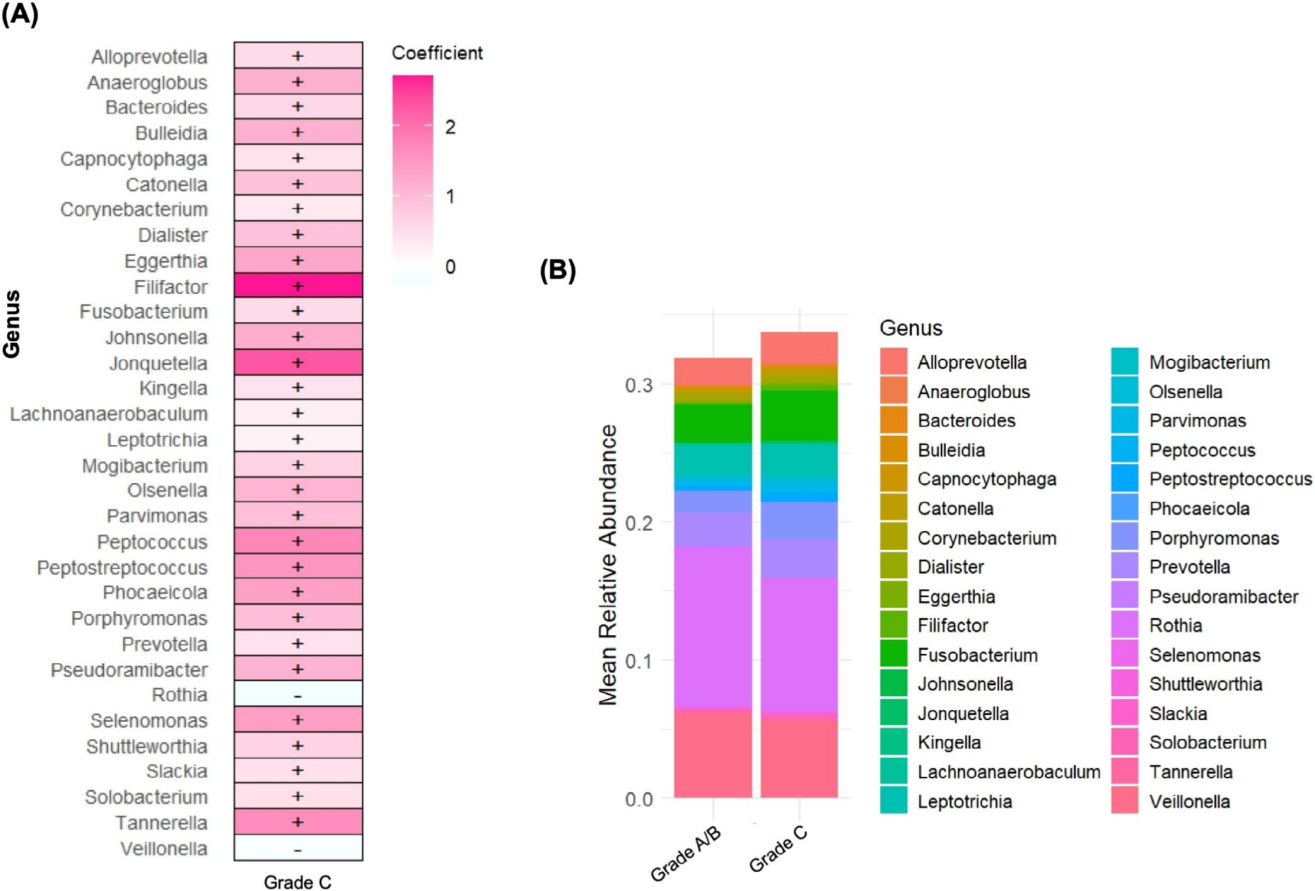
Differentially abundant taxa across different “periodontitis grades” (based on HOMD‐matching). (A) Differential abundance analysis using MaAsLin2. The heatmap presents genera significantly associated with Grade C periodontitis compared to the reference group (Grades A/B periodontitis). Effect sizes indicate the strength and direction of associations; “+” denotes positive and “–” negative associations (*p* < 0.05 after FDR correction). (B) Heatmap of overlapping genera. This heatmap illustrates the relative abundance of genera that were commonly identified as differentially abundant between Grade C and Grades A/B periodontitis.

Grade C was positively associated with periodontitis‐related genera and negatively associated with *Rothia* and *Veillonella* (Figure 4A). The genera most strongly associated with Grade C were *Filifactor* and *Jonquetella*. Figure 4B illustrates the relative abundance of genera across periodontitis grades: total HOMD‐based microbial abundance was higher in Grade C compared to Grades A/B. Sensitivity analyses with MaAsLin2 using the original SILVA v123 database assignment are shown in Figure S3. The results were consistent with the main findings, although additional genera, such as *Defluviitaleaceae_UCG‐011*, were also associated with Grade C.

### 3.3 Oral Microbiome Shifts Across Number of PPD ≥ 6 mm

**FIGURE 5:**
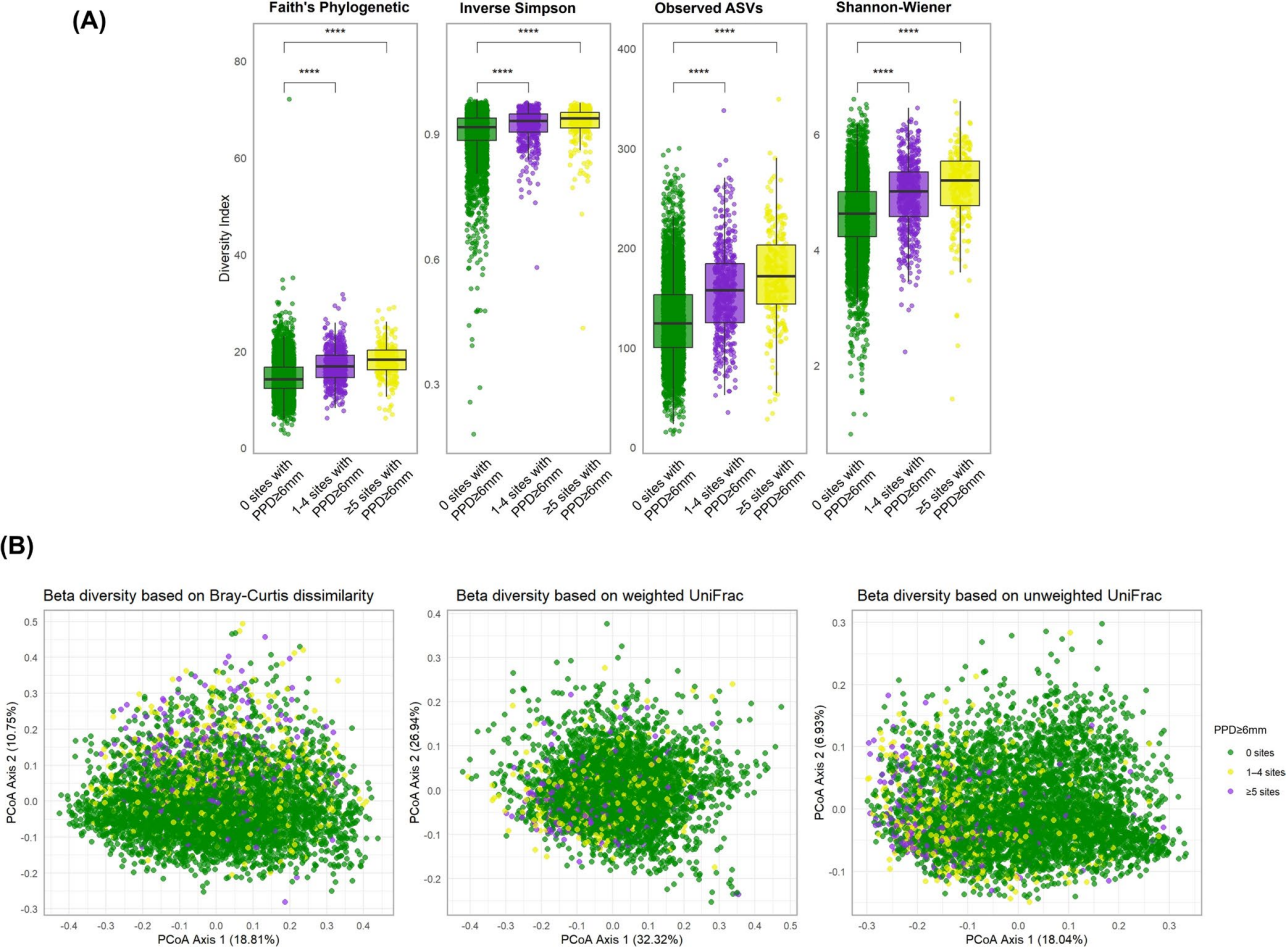
Alpha and beta diversity across groups defined by the “number of sites with PPD ≥ 6 mm”. (A) Alpha diversity indices. Box plots illustrate the interquartile range (IQR) from the 25th (Q1) to the 75th percentile (Q3), with the centerline indicating the median (Q2). Jittered points represent individual data points to visualize distribution density. Whiskers extend to 1.5× the IQR, and points beyond these are plotted as outliers. (****, *p* < 0.0001 after FDR correction). (B) Beta diversity indices. Principal Coordinates Analysis (PCoA) plots display the proportion of variance explained by the first two principal coordinates (PCo1 and PCo2), as indicated on the axes. Each point represents an individual sample, and spatial proximity indicates higher similarity in microbial community composition.

Alpha diversity increased proportionally with the number of sites with PPD ≥ 6 mm in both unadjusted and adjusted analyses (Figure 5A and Table 2). PERMANOVA indicated modest (0.4%–0.6%), yet often statistically significant, differences in beta diversity across groups defined by the number of sites with PPD ≥ 6 mm, while PERMDISP showed no significant differences in dispersion for any metric (Figure 5B and Table 3). Sensitivity analyses using survey‐weighted models yielded consistent results for both alpha and beta diversity (Tables S1 and S2).

**FIGURE 6:**
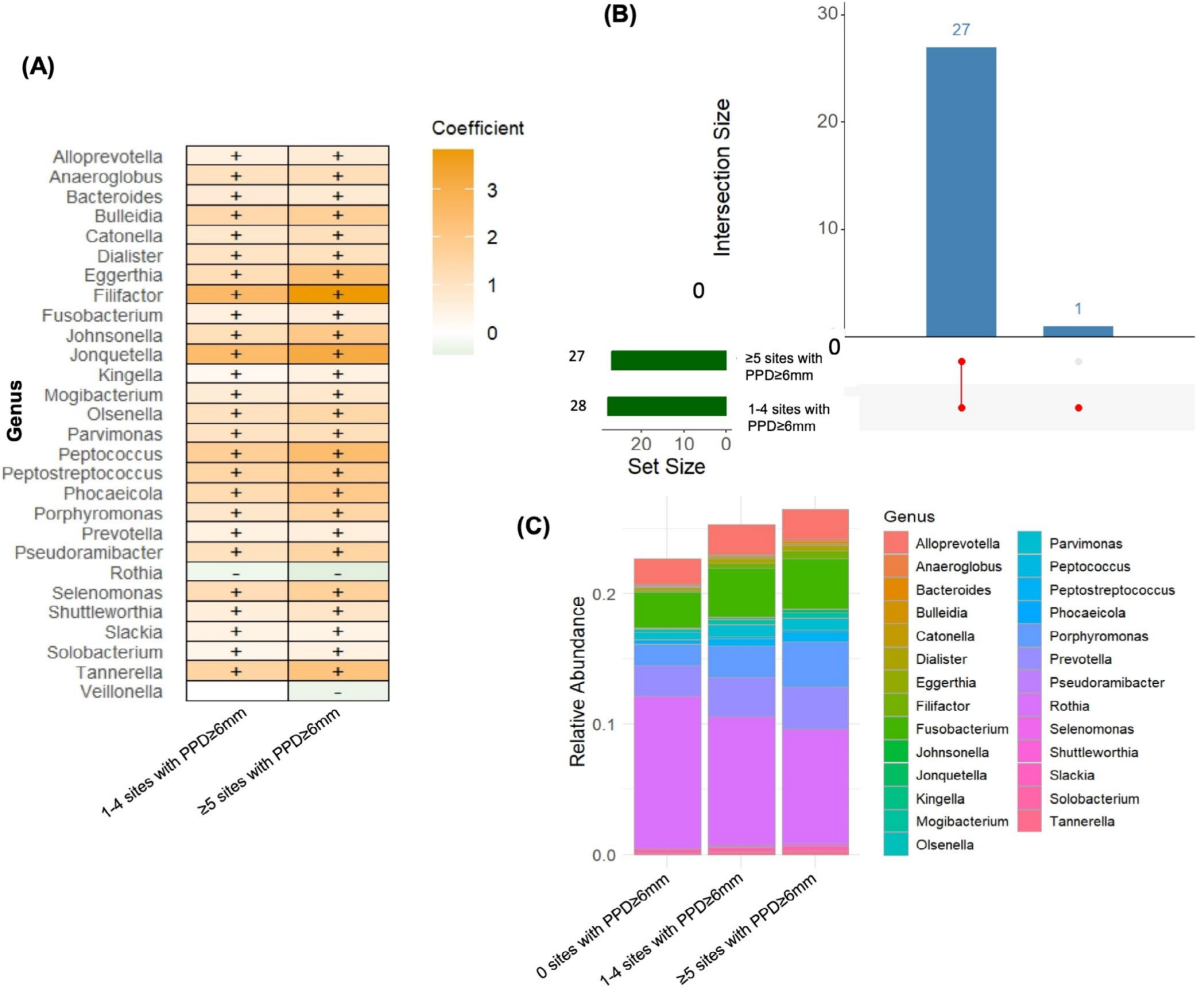
Differentially abundant taxa across groups defined by the “number of sites with PPD ≥ 6 mm” (based on HOMD‐matching). (A) Differential abundance analysis using MaAsLin2. The heatmap displays genera significantly associated with the presence of 1–4 or ≥ 5 sites with PPD ≥ 6 mm, compared to the reference group (0 sites with PPD ≥ 6 mm). Effect sizes indicate the strength and direction of associations; “+” denotes positive and “–” negative associations (*p* < 0.05 after FDR correction). (B) UpSet plot of differentially abundant genera. The horizontal bars indicate the total number of significantly altered genera in each group defined by the number of sites with PPD ≥ 6 mm. The vertical bars display the number of genera shared across combinations of groups. (C) Heatmap of overlapping genera. This heatmap illustrates the relative abundance of genera that were commonly identified as differentially abundant across the three groups defined by the number of sites with PPD ≥ 6 mm.

An increasing number of sites with PPD ≥ 6 mm was generally associated with enrichment of established periodontitis‐related genera and depletion of *Rothia* and *Veillonella* (Figure 6A). The genera most strongly associated with a higher number of deep pockets were *Filifactor* and *Jonquetella*. A total of 27 genera were commonly differentially abundant in both the 1–4 and ≥ 5 sites with PPD ≥ 6 mm groups, compared with individuals with 0 sites (Figure 6B). Figure 6C illustrates the relative abundance of these genera across the three PPD ≥ 6 mm groups. Total HOMD‐based microbial abundance showed a stepwise increase corresponding to higher numbers of sites with PPD ≥ 6 mm. Sensitivity analyses with MaAsLin2 using the original SILVA v123 database assignment are shown in Figure S2 and yielded consistent results, although additional genera, including *Defluviitaleaceae_UCG‐011*, were also enriched with increasing numbers of sites with PPD ≥ 6 mm.

## 4 Discussion

Findings from this population‐based study indicate that within‐sample microbial diversity (alpha diversity) increases with the severity, extent, and grade of periodontitis, peaking in Stage III generalized periodontitis. In Stage IV periodontitis, however, extensive tooth loss is associated with a decline in alpha diversity compared to Stage III generalized cases. This pattern is further accentuated in edentulous individuals, where alpha diversity falls below levels observed in participants without periodontitis. Between‐sample microbial variation (beta diversity) showed only subtle differences across periodontitis severity, extent, and grade. Nonetheless, within‐group dispersion tended to increase slightly in Stage IV periodontitis and in edentulism. Taxonomically, increasing severity, extent, and grade of periodontitis were associated with enrichment of established periodontitis‐related genera and *Jonquetella*, along with depletion of health‐related genera. Findings were consistent when the number of sites with PPD ≥ 6 mm was used as the outcome. In edentulous individuals, 32 genera were differentially abundant compared to those with no or localized Stage I–II periodontitis, 13 of which were also commonly altered in Stage III–IV periodontitis.

Unlike other chronic dysbiotic diseases–such as inflammatory bowel disease or atopic dermatitis–which are typically associated with reduced microbial diversity, the increasing severity of periodontitis paradoxically exhibited higher alpha diversity compared to periodontal health, in line with previous reports [50]. This finding, coupled with the minimal variance in overall oral microbiome composition explained by periodontal status, raises intriguing questions about the etiopathogenesis of periodontitis. It may, indeed, support the hypothesis that periodontitis is not primarily of microbial origin [51]. Rather, the disease process may alter the local environment in ways that promote the growth of a more diverse and heterogeneous microbial community. In diseases of microbial origin, one would indeed typically expect a few specific pathogenic species to dominate within samples and be consistently present across individuals. In the most severe forms of periodontitis, however, although certain genera are frequently detected, individual samples are more heterogeneous.

According to this model, the increased alpha diversity—particularly evident in Stage III, especially in its generalized form—may result from impaired host immune surveillance during disease, which permits the expansion of microbial species that would otherwise be constrained under homeostatic conditions [52]. Additionally, the inflammatory milieu characteristic of periodontitis likely creates a nutrient‐rich subgingival environment that supports the proliferation and coexistence of diverse taxa [52]. This is reflected in the pronounced increase in the number of differentially abundant genera observed in Stage III. The generalized form of Stage III likely amplifies this phenomenon by offering a broader range of ecological niches, including an increased number of deep periodontal pockets and more pronounced anaerobic conditions. This interpretation aligns with the observed proportional increase in alpha diversity with the number of sites exhibiting PPD ≥ 6 mm. However, it is important to acknowledge that elevated alpha diversity may also be compatible with the “ecological plaque hypothesis” [53] or reflect a byproduct of the increased total microbial biomass observed with advancing stages, extents, and grades of periodontitis, as more mature biofilms can support a greater variety of microbial species.

In terms of taxonomic composition, the present study showed how increasing severities, extents, and grades of periodontitis are associated with a significant enrichment of established periodontitis‐related genera—such as *Dialister*, *Filifactor, Fusobacterium*, *Porphyromonas*, *Prevotella*, and *Tannerella—*accompained by a relative decline, though not complete disappearance, of health‐related genera, including *Rothia* and *Veillonella*. These findings align with previous studies reporting the persistence of certain taxa across both health and disease states, further reinforcing the notion that periodontitis may be characterized by complex, overlapping microbial dynamics driven by environmental changes, rather than a simple, binary microbial shift that determines the disease itself [21, 54, 55]. By contrast, *Jonquetella* and *Defluviitaleaceae_UCG‐011*—the latter detected only in SILVA‐based sensitivity analyses—emerged among the genera most strongly associated with disease, despite not being traditionally classified as periodontitis‐related. While no previous studies have reported an association with *Jonquetella*, the observation related to *Defluviitaleaceae_UCG‐011* is not entirely novel: recent studies have described its enrichment in periodontal pockets and a potential association with greater disease severity [56, 57].

In contrast to periodontitis, edentulous individuals exhibited a marked reduction in within‐sample diversity and an increase in within‐group dispersion, despite no significant shift in centroids representing average bacterial composition. The observed decline in alpha diversity is consistent with previous studies and likely reflects microbial depletion following the loss of tooth surfaces and associated subgingival biofilms, which typically sustain diverse and structured microbial communities in the oral cavity [58]. Conversely, the increased within‐group dispersion may stem from the more heterogeneous oral environments characteristic of edentulism—including diverse etiologies of tooth loss (e.g., caries vs. periodontitis), variability in prosthetic rehabilitation, and a microbiome more directly influenced by recent food intake due to the absence of stable tooth‐associated niches. Despite the tooth‐loss‐related microbial depletion, the present analysis identified 13 microbial genera that were commonly differentially abundant in both Stage III–IV periodontitis and edentulism. The persistence of periodontitis‐associated taxa even in the absence of teeth may have relevant implications for implant‐based rehabilitation and the prevention of peri‐implant diseases [31, 59, 60, 61].

This study represents the first large‐scale, population‐based investigation of the oral microbiome in relation to periodontitis and edentulism. Strengths include a low risk of selection bias and the use of full‐mouth oral and periodontal examinations, which minimize the potential for information bias in outcome assessment—even though the retrospective application of the AAP/EFP classification framework (ACES) introduces some inherent limitations in capturing stages and grades of periodontitis [41]. In addition, to enhance interpretation and facilitate integration with the existing literature, significant genera identified in the SILVA v123–based analysis were subsetted and cross‐referenced with the HOMD, and both sets of classifications were reported. SILVA and HOMD differ fundamentally in scope and curation. HOMD is specifically curated for human oral taxa, with high‐resolution annotation of known oral species, making it particularly reliable for detecting classical oral bacteria and pathogens. However, the focused scope of HOMD may omit less well‐characterized or recently described taxa. For instance, *Defluviitaleaceae_UCG‐011*, which has been associated with periodontitis in the present study and in previous reports [56, 57], is absent from HOMD but included in SILVA's broader database. Although Scope mouthwash contains antiseptic agents such as cetylpyridinium chloride and alcohol, the use of 16S rRNA gene sequencing—which targets bacterial DNA rather than viable cells—mitigates concerns about potential bias introduced by antimicrobial components. A recent study indeed reported no significant differences in DNA yield, alpha diversity, or genus‐level taxonomic composition between Scope‐collected oral rinses and unstimulated saliva [62], supporting the validity of this sampling method for oral microbiome profiling.

Nevertheless, readers should be aware of potential unmeasured residual confounding (e.g., related to different prosthetic rehabilitations, plaque levels, or history of periodontal treatment), as well as the relatively low taxonomic resolution of the microbiome assessment method used (16S rRNA sequencing with a 125 bp region), which also precluded the evaluation of nonbacterial microorganisms. Genus‐level analyses may obscure important biological variation, as different species within the same genus can differ markedly in virulence and pathogenic potential [63]. For instance, *Porphyromonas catoniae* is a common and benign oral colonizer in infants [64], whereas *P. gingivalis* has been implicated as a keystone pathogen in periodontitis [65]. Even within *P. gingivalis* , virulence varies significantly across strains [66]. Moreover, while oral rinse samples have been shown to correlate with subgingival plaque samples [67] and may better reflect the current conceptualization of periodontitis as a systemic disease [30], their broader representation of microorganisms from multiple oral sites may underestimate the impact of periodontitis compared to their localized enrichment in subgingival biofilms. Finally, although sample weights were applied to alpha diversity and PERMANOVA analyses, currently there is a lack of validated pipelines for incorporating survey weights into microbiome analyses involving taxonomic composition or differential abundance testing. Consequently, weighted analyses were presented only as sensitivity analyses. Where analyses for complex samples could be applied, limited differences between weighted and unweighted results were, however, observed. Given the absence of robust, validated methods for applying survey weights to abundance‐based analyses, exploratory or ad hoc approaches were deliberately avoided to prevent introducing bias or reducing interpretability. This limitation underscores the methodological need for the development of new analytical frameworks capable of reliably integrating complex survey designs across the full microbiome analysis pipeline in population‐based studies.

Advancements in the field will require building on these findings through shotgun metagenomic and functional analyses to capture greater microbial and functional resolution. Moreover, the generalizability of these results to populations beyond the U.S. remains to be established. Finally, given the cross‐sectional design, causal relationships cannot be inferred—leaving unresolved, despite the speculations discussed here, one of the most fundamental questions in periodontology: whether the oral microbiome is a cause or a consequence of periodontitis [51].

## 5 Conclusions

This population‐based study shows that periodontitis is characterized by a progressive increase in alpha diversity as stage, extent, and grade advance, accompanied by only subtle shifts in beta diversity. However, this alpha diversity trend reverses with extensive tooth loss (Stage IV) and edentulism. Taxonomically, increasing severity, extent, and grade of periodontitis are associated with enrichment of established periodontitis‐related genera and *Jonquetella*, alongside depletion of health‐related genera. The persistence of periodontitis‐associated taxa even in the absence of teeth may carry important implications for implant‐based rehabilitations and the prevention of peri‐implant diseases.

## Author Contributions

P.B. contributed to study design, data analysis, and manuscript drafting. F.R.M.L. contributed to study design, data interpretation, and critically revised the manuscript. J.R.H.T. contributed to study design, data interpretation, and manuscript drafting. J.H. contributed to data analysis and critically revised the manuscript. G.G.N. contributed to study design, data interpretation, and critically revised the manuscript. M.R. contributed to study conception and design, data interpretation, and manuscript drafting.

## Conflicts of Interest

The authors declare no conflicts of interest related to this study, which was self‐funded. Mario Romandini serves as Editor‐in‐Chief and Gustavo Nascimento as Associate Editor of the *Journal of Periodontal Research*, and they are also authors of this article. In accordance with Wiley's standard policies for submissions by Editors, they were excluded from the editorial decision‐making and remained blinded throughout the peer review process, with another journal Editor designated as acting Editor‐in‐Chief.

## Supporting information


**Table S1:** Sensitivity analysis (survey‐weighted): Alpha diversity indices across different “periodontal status,” “periodontitis grades” and “number of sites with PPD ≥ 6 mm.”
**Table S2:** Sensitivity analysis (survey‐weighted): Beta diversity across different “periodontal status,” “periodontitis grades” and “number of sites with PPD ≥ 6 mm.”

**Figure S1:** Directed Acyclic Graph (DAG) showing hypothesized relationships between oral microbiome, confounders and periodontal status/periodontitis grading/number of sites with PPD ≥ 6 mm.

**Figure S2:** Differentially abundant taxa across different “periodontal status” categories (based on SILVA v123 database).

**Figure S3:** Differentially abundant taxa across different “periodontitis grades” categories (based on SILVA v123 database).

**Figure S4:** Differentially abundant taxa across groups defined by the “number of sites with PPD ≥ 6 mm” categories (based on SILVA v123 database).

## Data Availability

The data that support the findings of this study are openly available in the National Centre for Health Statistics, Centers for Disease Control and Prevention, at https://wwwn.cdc.gov/nchs/nhanes/.
