# Supplementary material for: Oral Microbiome Signatures in Periodontitis and Edentulism—A Population‐Based Study
Source: J Periodontal Res. 2025 Nov 1;60(11):1101–16. doi: 10.1111/jre.70046 (PMC12779175; doi:10.1111/jre.70046)
Supplement: Supplementary file 1 — Table S1: Sensitivity analysis (survey‐weighted): Alpha diversity indices across different “periodontal status,” “periodontitis grades” and “number of sites with PPD ≥ 6 mm.” Table S2: Sensitivity analysis (survey‐weighted): Beta diversity across different “periodontal status,” “periodontitis grades” and “number of sites with PPD ≥ 6 mm.” [file JRE-60-1101-s004.docx]

**Table S1.** Sensitivity analysis (survey-weighted): Alpha diversity indices across different “periodontal status”, “periodontitis grades” and “number of sites with PPD ≥6mm”.

| **Alpha Diversity Index** | **Periodontal outcome** | **Estimate** | **Standard error** | **p-value** |
| --- | --- | --- | --- | --- |
| **Faith’s Phylogenetic Diversity** | No/Stage I-II localized | *Reference* | | |
|  | Stage I-II Generalized | 0.143 | 0.137 | 0.464 |
|  | Stage III Localized | 1.187 | 0.226 | **<0.001** |
|  | Stage III Generalized | 2.941 | 0.396 | **<0.001** |
|  | Stage IV | 1.777 | 0.257 | **<0.001** |
|  | Edentulism | -1.451 | 0.511 | **0.009** |
|  | Grade A/B | *Reference* | | |
|  | Grade C | 2.078 | 0.228 | **<0.001** |
|  | 0 sites with PPD≥6mm | *Reference* | | |
|  | 1-4 sites with PPD≥6mm | 2.089 | 0.211 | **<0.001** |
|  | ≥5 sites with PPD≥6mm | 3.233 | 0.224 | **<0.001** |
| **Inverse Simpson** | No/Stage I-II Localized | *Reference* | | |
|  | Stage I-II Generalized | -0.002 | 0.002 | 0.374 |
|  | Stage III Localized | 0.004 | 0.003 | 0.252 |
|  | Stage III Generalized | 0.02 | 0.004 | **<0.001** |
|  | Stage IV | 0.014 | 0.005 | **0.013** |
|  | Edentulism | -0.027 | 0.013 | 0.098 |
|  | Grade A/B | *Reference* | | |
|  | Grade C | 0.013 | 0.003 | **0.001** |
|  | 0 sites with PPD≥6mm | *Reference* | | |
|  | 1-4 sites with PPD≥6mm | 0.014 | 0.004 | **0.002** |
|  | ≥5 sites with PPD≥6mm | 0.014 | 0.005 | **0.022** |
| **Observed ASVs** | No/Stage I-II Localized | *Reference* | | |
|  | Stage I-II Generalized | 0.602 | 1.608 | 0.803 |
|  | Stage III Localized | 18.359 | 3.037 | **<0.001** |
|  | Stage III Generalized | 43.87 | 6.289 | **<0.001** |
|  | Stage IV | 28.208 | 3.277 | **<0.001** |
|  | Edentulism | -14.358 | 6.018 | **0.032** |
|  | Grade A/B | *Reference* | | |
|  | Grade C | 26.602 | 2.874 | **<0.001** |
|  | 0 sites with PPD≥6mm | *Reference* | | |
|  | 1-4 sites with PPD≥6mm | 25.571 | 2.622 | **<0.001** |
|  | ≥5 sites with PPD≥6mm | 38.094 | 3.012 | **<0.001** |
| **Shannon-Wiener** | No/Stage I-II Localized | *Reference* | | |
|  | Stage I-II Generalized | 0.003 | 0.019 | 0.908 |
|  | Stage III Localized | 0.153 | 0.032 | **<0.001** |
|  | Stage III Generalized | 0.492 | 0.065 | **<0.001** |
|  | Stage IV | 0.289 | 0.051 | **<0.001** |
|  | Edentulism | -0.338 | 0.114 | **0.009** |
|  | Grade A/B | *Reference* | | |
|  | Grade C | 0.308 | 0.045 | **<0.001** |
|  | 0 sites with PPD≥6mm | *Reference* | | |
|  | 1-4 sites with PPD≥6mm | 0.304 | 0.047 | **<0.001** |
|  | ≥5 sites with PPD≥6mm | 0.426 | 0.049 | **<0.001** |

*Footnote:*

Bold indicates statistical significance (p<0.05)

**Table S2.** Sensitivity analysis (survey-weighted): Beta diversity across different “periodontal status”, “periodontitis grades and “number of sites with PPD ≥6mm”.

|  |  | PERMANOVA | |
| --- | --- | --- | --- |
| Beta diversity metric | **Periodontal outcome** | **F. Model** | **R^2^ (%)** |
| Bray Curtis Dissimilarity | No/Stage I-II Localized | *Reference* | |
|  | Stage I-II Generalized | 2.293 | 0.071 |
|  | Stage III Localized | 7.995 | 0.331 |
|  | Stage III Generalized | 18.382 | 1.070 |
|  | Stage IV | 7.529 | 0.316 |
|  | Edentulism | 3.367 | 0.190 |
|  | Grade A/B | *Reference* | |
|  | Grade C | 43.914 | 0.823 |
|  | 0 sites with PPD≥6mm | *Reference* | |
|  | 1-4 sites with PPD≥6mm | 14.087 | 0.279 |
|  | ≥5 sites with PPD≥6mm | 20.242 | 0.422 |
| Weighted Unifrac | No/Stage I-II Localized | *Reference* | |
|  | Stage I-II Generalized | 3.148 | 0.097 |
|  | Stage III Localized | 9.259 | 0.381 |
|  | Stage III Generalized | 24.468 | 1.417 |
|  | Stage IV | 9.019 | 0.378 |
|  | Edentulism | 1.771 | 0.097 |
|  | Grade A/B | *Reference* | |
|  | Grade C | 52.218 | 0.976 |
|  | 0 sites with PPD≥6mm | *Reference* | |
|  | 1-4 sites with PPD≥6mm | 23.755 | 0.469 |
|  | ≥5 sites with PPD≥6mm | 27.120 | 0.565 |
| Unweighted Unifrac | No/Stage I-II Localized | *Reference* | |
|  | Stage I-II Generalized | 1.714 | 0.052 |
|  | Stage III Localized | 20.831 | 0.839 |
|  | Stage III Generalized | 28.150 | 1.604 |
|  | Stage IV | 16.879 | 0.699 |
|  | Edentulism | 6.751 | 0.372 |
|  | Grade A/B | *Reference* | |
|  | Grade C | 125.246 | 2.298 |
|  | 0 sites with PPD≥6mm | *Reference* | |
|  | 1-4 sites with PPD≥6mm | 34.225 | 0.666 |
|  | ≥5 sites with PPD≥6mm | 35.084 | 0.720 |

**Figure S1.** Directed Acyclic Graph (DAG) showing hypothesized relationships between oral microbiome, confounders and periodontal status/periodontitis grading/number of sites with PPD≥6 mm.

**Figure S2.** Differentially abundant taxa across different “periodontal status” categories (based on SILVA v123 database).

*Footnote:*
**(A)** Differential abundance analysis using MaAsLin2. The heatmap presents genera significantly associated with each periodontal status compared to the reference group (No periodontitis / Stage I–II localized). Effect sizes indicate the strength and direction of associations; “+” denotes positive and “–” negative associations (p < 0.05 after FDR correction).

**(B)** UpSet plot of differentially abundant genera*.* The horizontal bars indicate the total number of significantly altered genera in each periodontal status group. The vertical bars display the number of genera shared across combinations of groups.

**(C)** Heatmap of overlapping genera*.* This heatmap illustrates the relative abundance of genera that were commonly identified as differentially abundant across multiple periodontal status categories.

**Figure S3.** Differentially abundant taxa across different “periodontitis grades” categories (based on SILVA v123 database).

*Footnote:*
**(A)** Differential abundance analysis using MaAsLin2. The heatmap presents genera significantly associated with Grade C periodontitis compared to the reference group (Grades A/B periodontitis). Effect sizes indicate the strength and direction of associations; “+” denotes positive and “–” negative associations (p < 0.05 after FDR correction).

**(B)** Heatmap of overlapping genera*.* This heatmap illustrates the relative abundance of genera that were commonly identified as differentially abundant between Grade C and Grades A/B periodontitis.

**Figure S4.** Differentially abundant taxa across groups defined by the “number of sites with PPD ≥ 6 mm” categories (based on SILVA v123 database).

*Footnote:*
**(A)** Differential abundance analysis using MaAsLin2. The heatmap displays genera significantly associated with the presence of 1–4 or ≥5 sites with PPD ≥ 6 mm, compared to the reference group (0 sites with PPD ≥ 6 mm). Effect sizes indicate the strength and direction of associations; “+” denotes positive and “–” negative associations (p < 0.05 after FDR correction).

**(B)** UpSet plot of differentially abundant genera*.* The horizontal bars indicate the total number of significantly altered genera in each group defined by the number of sites with PPD≥6 mm. The vertical bars display the number of genera shared across combinations of groups.

**(C)** Heatmap of overlapping genera*.* This heatmap illustrates the relative abundance of genera that were commonly identified as differentially abundant across the three groups defined by the number of sites with PPD ≥ 6 mm.
