# Supplementary figures and images for: Oral Microbiome Signatures in Periodontitis and Edentulism—A Population‐Based Study
Source: J Periodontal Res. 2025 Nov 1;60(11):1101–16. doi: 10.1111/jre.70046 (PMC12779175; doi:10.1111/jre.70046)

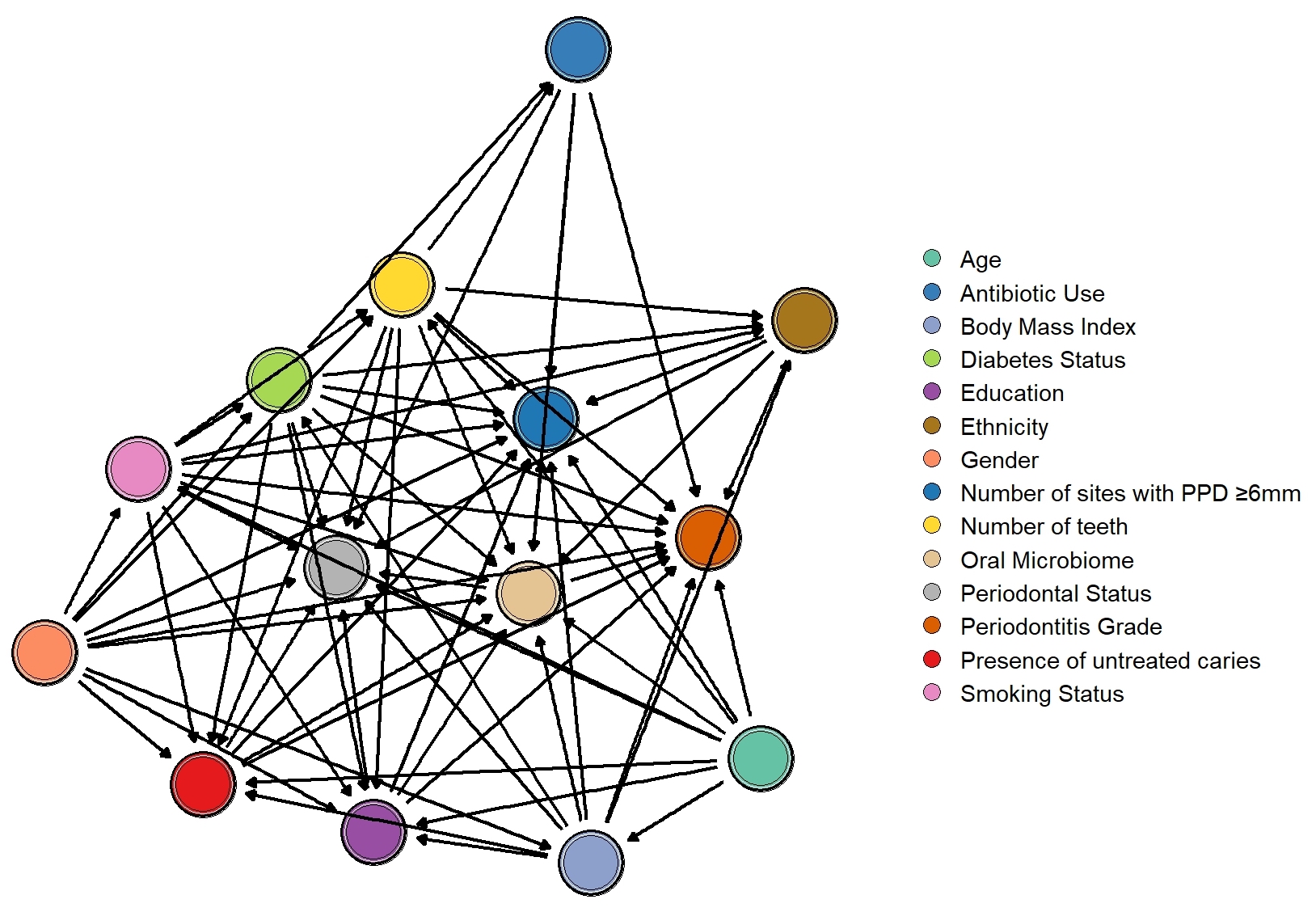

Supplement: Supplementary file 2 — Figure S1: Directed Acyclic Graph (DAG) showing hypothesized relationships between oral microbiome, confounders and periodontal status/periodontitis grading/number of sites with PPD ≥ 6 mm. [file JRE-60-1101-s001.jpeg]

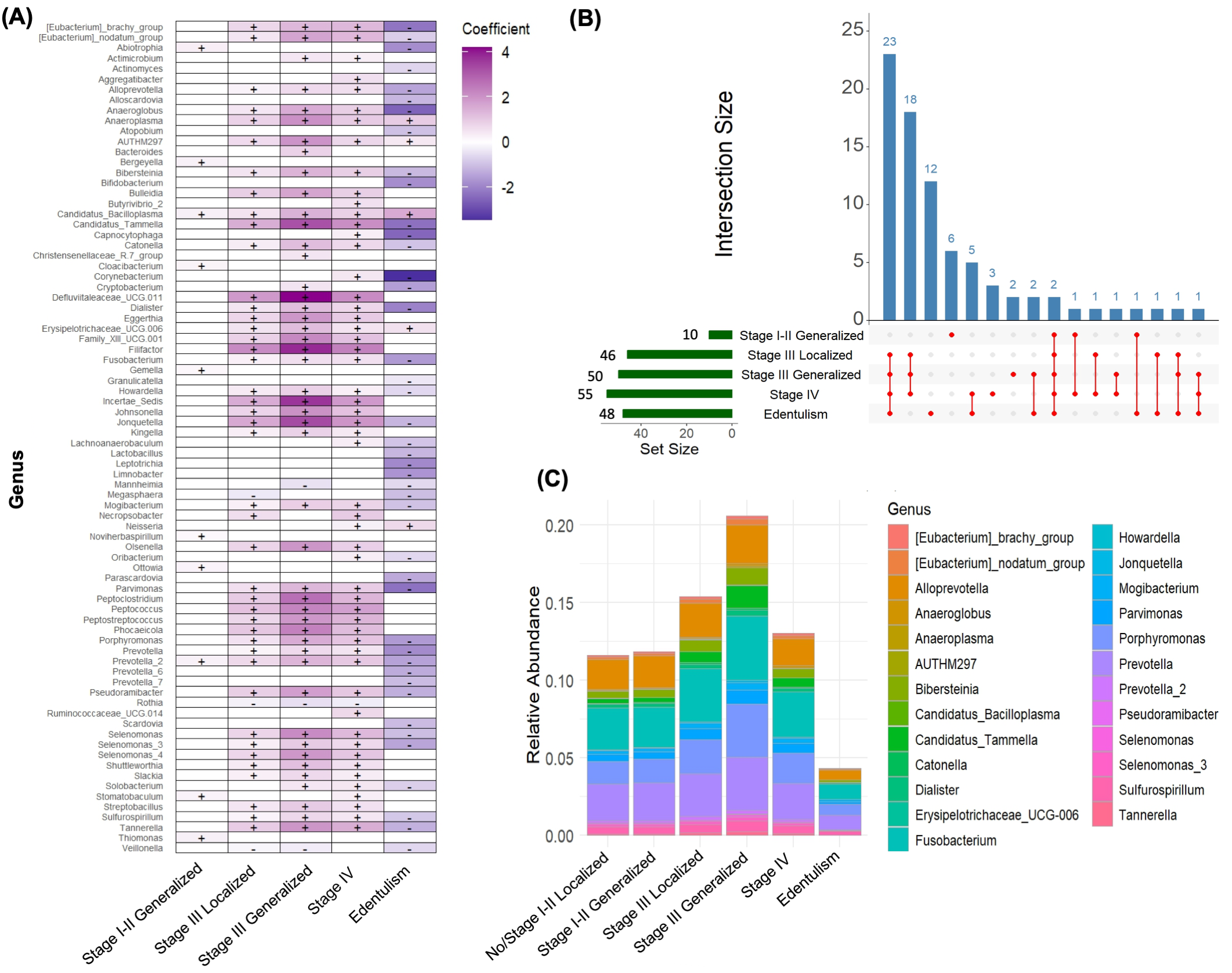

Supplement: Supplementary file 3 — Figure S2: Differentially abundant taxa across different “periodontal status” categories (based on SILVA v123 database). [file JRE-60-1101-s002.png]

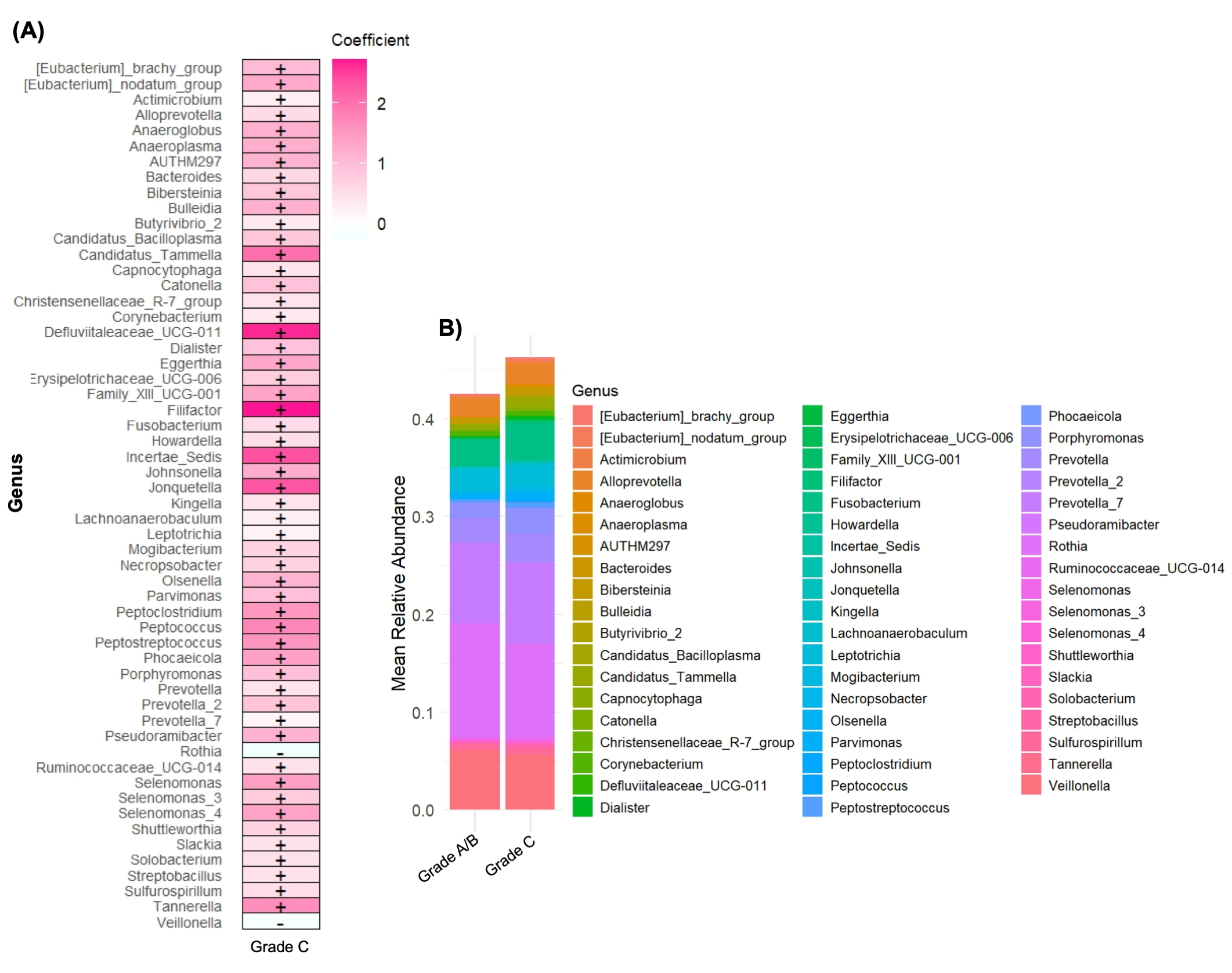

Supplement: Supplementary file 4 — Figure S3: Differentially abundant taxa across different “periodontitis grades” categories (based on SILVA v123 database). [file JRE-60-1101-s005.png]

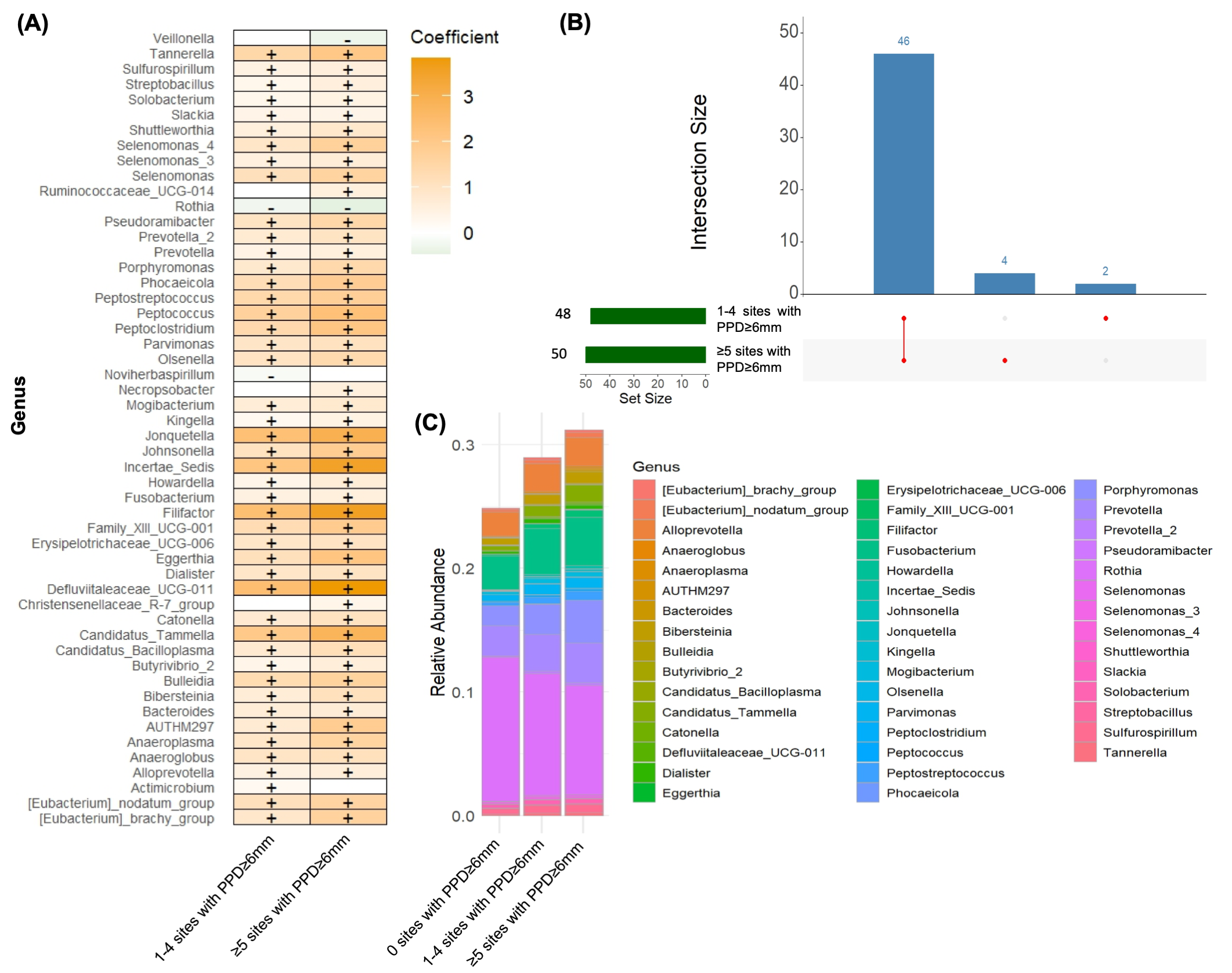

Supplement: Supplementary file 5 — Figure S4: Differentially abundant taxa across groups defined by the “number of sites with PPD ≥ 6 mm” categories (based on SILVA v123 database). [file JRE-60-1101-s003.png]
